# Adverse Events with Amiodarone—Will the List Ever End?

**DOI:** 10.19102/icrm.2018.090307

**Published:** 2018-03-15

**Authors:** James A. Reiffel

**Affiliations:** ^1^Department of Medicine, Division of Cardiology, Electrophysiology Section, Columbia University, New York, NY, USA

**Keywords:** Adverse effects, amiodarone, SIADH, toxicity

Some might say that amiodarone is a wonder drug. As an antiarrhythmic, it can be effective against supraventricular tachycardias, whether utilizing the atrioventricular node or not, atrial fibrillation, and a variety of ventricular tachyarrhythmias.^1,2^ In most active control studies, amiodarone has been more effective than the comparator drugs (including sotalol, propafenone, and more), although it commonly takes longer for its effect to occur due to its long elimination half-life. Moreover, in my personal experience, it is also incredibly potent in the treatment of angina pectoris and other symptomatic cardiac ischemic conditions—the target that prompted its original development.

Conversely, others may marvel moreso at its inconveniences, innumerable drug interactions, and long list of adverse effects—all of which serve to limit its utility as a first-line agent.^3^

In this issue of *The Journal of Innovations in Cardiac Rhythm Management*, Barham et al., in the format of a case report and a review of the literature,^4^ call attention to an infrequently reported adverse consequence of amiodarone use that I strongly suspect is not known to most practitioners or clinical pharmacologists; that is, marked hyponatremia, which most likely occurred due to the inducement of the syndrome of inappropriate secretion of antidiuretic hormone (SIADH). Their report details the time course as related to amiodarone use, both with respect to its appearance and its termination. As with the other rare reports of this association (specifically, the 17 cases mentioned in the review by Barham et al. and several more I identified^5,6^), underlying disorders, including heart failure, pulmonary disorders, and the like, have also been present. Nonetheless, with considered deliberation and evaluation, the relationship to amiodarone seems highly likely, though its development in the absence of other underlying pathologies is uncertain. Unfortunately, given the severity of the presentations and the underlying patient conditions, further confirmation with a rechallenge protocol has not typically been part of the evaluation process. Long-term follow-up data to confirm the absence of any recurrence while off amiodarone have also not been generally available. Additionally, the mechanism of the precipitation of SIADH by amiodarone remains inadequately explored. These shortcomings notwithstanding, ensuring physicians are aware of this additional potential of amiodarone seems worthwhile.

I hope that our readers appreciate becoming aware of this rare consequence of amiodarone as well as the *Journal*’s growing interest in cardiac rhythm pharmacology and that, moving forward, they will continue to submit relevant, interesting observations and clinical trial reports to share with their peers.
